# Soil carbon determination by thermogravimetrics

**DOI:** 10.7717/peerj.6

**Published:** 2013-02-12

**Authors:** Robert Pallasser, Budiman Minasny, Alex B. McBratney

**Affiliations:** Faculty of Agriculture and Environment, Department of Environmental Sciences, The University of Sydney, Australia

**Keywords:** Soil carbon, Organomineral complex, Loss on ignition, TGA/MS, Thermogravimetric analysis

## Abstract

Determination of soil constituents and structure has a vital role in agriculture generally. Methods for the determination of soil carbon have in particular gained greater currency in recent times because of the potential that soils offer in providing offsets for greenhouse gas (CO_2_-equivalent) emissions. Ideally, soil carbon which can also be quite diverse in its makeup and origin, should be measureable by readily accessible, affordable and reliable means. Loss-on-ignition is still a widely used method being suitably simple and available but may have limitations for soil C monitoring. How can these limitations be better defined and understood where such a method is required to detect relatively small changes during soil-C building? Thermogravimetric (TGA) instrumentation to measure carbonaceous components has become more interesting because of its potential to separate carbon and other components using very precise and variable heating programs. TGA related studies were undertaken to assist our understanding in the quantification of soil carbon when using methods such as loss-on-ignition. Combining instrumentation so that mass changes can be monitored by mass spectrometer ion currents has elucidated otherwise hidden features of thermal methods enabling the interpretation and evaluation of mass-loss patterns. Soil thermogravimetric work has indicated that loss-on-ignition methods are best constrained to temperatures from 200 to 430 °C for reliable determination for soil organic carbon especially where clay content is higher. In the absence of C-specific detection where mass only changes are relied upon, exceeding this temperature incurs increasing contributions from inorganic sources adding to mass losses with diminishing contributions related to organic matter. The smaller amounts of probably more recalcitrant organic matter released at the higher temperatures may represent mineral associated material and/or simply more refractory forms.

## Introduction

Soils are significant global reservoirs for carbon (C) and are therefore receiving a great deal of attention for their capacity to offset the increasing levels of atmospheric CO_2_. In addition there is the immense value of having higher amounts of organic matter (OM) for improved soil function and structure which have a positive outcome in terms of food security through resilience against degradation. To assess the effectiveness of land use practices where temporal changes in soil carbon building can be small, measurement instruments/methods need to be sufficiently sensitive and provide robust values. Added to this, soil carbon content can be fairly variable (Goidts, Van Wesemael & Crucifix, 2009) from one place to another and also occur in a variety of forms with different stabilities and therefore residence times (McCarthy et al., 2008 and references therein), a key issue for soil carbon storage. As a consequence, soil carbon analysis methods have become a lively area of interest, especially considering the need for the mapping of large areas and this inherent variability.

Dry combustion by elemental analysis of soils is often used to provide precise total carbon determinations on fairly small amounts sub-sampled. On the other hand the loss-on-ignition (LOI) method, which is widespread due to its low cost simplicity, allows larger amounts to be tested appropriate to the scale of the task at hand. However the technique has attracted a great deal of discussion about its accuracy and equally OM to C conversion factors have been controversial (Pribyl, 2010; Sutherland, 1998 and others). By this technique, previously dried and weighed soils are heated in a muffle furnace to obtain the mass of material lost which can be transformed to % carbon by reported conversion factors. More accurate factors can be derived from a ‘calibration’ set of soils (via elemental analysis) from the study area which take account of local conditions but there is no set protocol for temperatures (can be 500 °C plus) and durations which vary between soil laboratories.

Thermogravimetric analysis (TGA) is a method traditionally used for the mineral (clay and oxides) components of soils, generally after the removal of OM. Although the variety of carbon forms present in soils, which can range in their kinetic behaviour, can also be broadly distinguished (and quantified) using controlled heating rates (see examples in Laird et al., 2008). For example CaCO_3_ which begins to degrade after 600 °C, is readily separated from organic-C (Kasozi, Nkedi-Kizza & Harris, 2009). Thus the thermal distribution of all components in a whole soil, including those that are carbon-bearing, can provide a fingerprint characterisation as well as quantitative information. Applications of this method to tease out differences within the organic fraction in soils have been relatively small but a number of researchers (notable examples Dell’Abate, Benedetti & Sequi, 2000; Dell’Abate, Benedetti & Brookes, 2003; Siewert, 2004; Manning, Lopez-Capel & Barker, 2005; references in the review by Plante, Fernandez & Leifeld, 2009) have more recently reported TGA related investigations on either soils or composted materials.

In this study TGA and related techniques were applied to a range of whole soils to obtain a better understanding of SOM thermal distribution and what this means for loss-on-ignition methods. Inorganic reactions were not followed up in detail here but instead were an overall consideration when evaluating thermal methods used to measure organic matter. A database containing comprehensive information on LOI, carbon and texture was also analysed to support the observations from thermal studies and to indicate the influence of clay content when measuring C using LOI methods.

## Materials and Methods

### Collection and preparation of soil subsamples

A cross-section of soils (36) were obtained from different areas in NSW, Australia (Table 1). Soils were extracted from the field using hand or mechanized corer and intervals removed to plastic tubes for transport and storage. Some of the soils included here were collected as part of other studies hence some variation in depths sampled (indicated on Table 1). Representative amounts were sub-sampled for replicate analyses (>2 per site) to confirm differences observed for these localities which varied considerably by soil type and the amount of OM present. Samples were air and oven (40 °C) dried prior to removal of recent organic matter such as visible root material by passing a 2 mm sieve (McKenzie et al., 2000). For elemental and thermogravimetric analysis samples were then ground by mortar and pestle and dry sieved (<100 µm) to obtain homogenous material to facilitate the small sample loadings. In addition some soil references (Rayment et al., 2007) acquired through Proficiency Services Ltd. Hamilton, NZ were included in this study. A legacy dataset of soils (CSIRO National Soil Database) containing LOI, dry combustion and textural data (methods according to Rayment & Lyons, 2011) was also used.

**Table 1:** List of soils used for these experiments detailing mass losses from thermal analysis.

Soil and origin	Landuse	Depth (cm)	% C	% mass losses		
				200–430 °C	430–590 °C	590–750 °C
Pilliga chromosol 2	Crop	Surface	0.2	0.7	1.9	
Hunter Valley Dermosol 3	Mixed farming	30–60	0.2	0.9	2.7	
Hunter Valley Dermosol 2	Mixed farming	30–60	0.2	0.9	2.8	0.7
Hunter Valley Dermosol 5	Mixed farming	30–60	0.2	0.7	2.3	
Hunter Valley Dermosol 4b	Mixed farming	60–100	0.2	0.8	1.7	9.8
Lansdowne Kandosol	Mixed farming	0–5	0.2	0.4	0.3	
Hunter Valley Dermosol 4a	Mixed farming	30–60	0.3	1.4	3.0	
Pilliga chromosol 1	Crop	Surface	0.4	1.1	3.1	8.0
Namoi Vertisol 2	Crop	10–30	0.5	1.1	2.5	
Narrabri Vertisol 1b	Crop	16–30	0.6	0.9	1.5	
Camden 2c	Lucerne	21–30	0.8	1.3	3.1	
Hunter Valley Dermosol 8c	Mixed farming	30–60	0.8	0.4	3.4	
Camden 2b	Lucerne	11–20	0.8	1.4	2.9	
Narrabri Vertisol 2b	Crop	16–30	0.8	1.4	2.2	
Narrabri Vertisol 1a	Crop	0–15	0.9	2.1	2.4	
Camden 3c	Lucerne	21–30	0.9	1.4	3.1	
Narrabri Vertisol 2a	Crop	0–15	1.0	1.5	1.5	
Soil standard 1	Reference material	Unknown	1.0	1.3	3.8	1.9
Hunter Valley Dermosol 1d	Mixed farming	30–60	1.0	3.7	4.2	2.5
Hunter Valley Dermosol 8b	Mixed farming	15–30	1.1	0.6	3.7	
Namoi Vertisol 1	Crop	0–10	1.4	2.2	2.0	
Hunter Valley Dermosol 7a	Mixed farming	0–5	1.5	1.3	4.2	1.7
Hunter Valley Dermosol 1c	Mixed farming	15–30	1.5	2.5	3.7	0.6
Liverpool Plains Vertisol 2	Crop	Surface	1.6	3.1	4.1	
Hunter Valley Dermosol 8a	Mixed farming	5–15	1.7	0.9	1.9	
Camden 3b	Lucerne	11–20	1.7	2.1	2.7	
Liverpool Plains Vertisol 1	Pasture	Surface	1.8	3.3	4.8	
Hunter Valley Dermosol 7b	Mixed farming	15–30	1.8	1.4	3.5	
Camden 2a	Lucerne	0–10	1.8	2.2	2.5	
Camden 1	Lucerne	0–30	2.0	2.0	2.3	
Camden 3a	Lucerne	0–10	2.3	3.1	3.1	
Hunter Valley Dermosol 6	Mixed farming	25	2.4	2.7	2.6	tr
Soil standard 2	Reference material	Unknown	2.6	4.5	1.7	
Hunter Valley Dermosol 1b	Mixed farming	5–15	2.6	3.6	5.7	
Hunter Valley Dermosol 1a	Mixed farming	0–5	3.4	6.2	6.6	
Soil standard 3	Reference material	Unknown	4.0	7.5	2.0	
Sydney Basin Kandosol	Vegetated	0–5	6.1	9.5	1.5	
Sydney Basin loam	Horticulture	0–5	9.5	13.8	7.1	1.1
Sydney Basin sandy loam	Horticulture	0–5	9.6	15.8	6.3	1.2

### Measurement of carbon by dry combustion using elemental analysis (EA)

Carbon concentrations were determined using a Vario-max Elementar CN analyser (Hanau, Germany) with 900 °C combustion temperatures (for detailed explanation of the dry combustion method, refer to Rayment & Lyons, 2011).

### Thermogravimetric analysis (TGA)

Pure TGA experiments were conducted on a TA Instruments 2950 thermogravimetric analyser which can heat up to 100 mg of material at variably programmable rates to over 1000 °C. Typically 60–100 mg of soil (equates to several mg OM) were suspended in a platinum pan (tare weight 360 mg) such that comparable amounts of C (ranging from <1% to 15%) were analysed. The incremental mass changes (resolution of 0.0001 mg) recorded over this program were processed using TA Universal Analysis 2000 software.

An oxygen-rich atmosphere was used (60  ml/min O_2_ purging furnace and 40  ml/min N_2_ purging balance) as it provides O_2_ in excess for SOM conversion to CO_2_ which would otherwise degrade in an inert stream and potentially leave a charred residue leading to an incomplete mass loss (possible ghost signals) and underestimation. Evolution of non-combustible components such as interlayer water and OH^−^ units or CO_2_ from carbonates are unaffected by the type of atmosphere used during thermal decomposition. Samples were heated to 200 °C, held for 10 min (surface dehydration) and then ramped at 10 °C/min to 700 °C and finally held for another 10 min. Carbonatic soils were heated at the same rate but beyond 700 °C until completely calcined. Heating rates of 10 °C/min from 200 to 600 °C provided the optimal resolution in soils.

### Thermogravimetric analysis/mass spectrometry (TGA/MS)

To look more deeply into the various mass change events, thermal analyses on a small subset of soils were run in combination with mass spectrometry. TGA/MS was carried out with a Setaram setsys 16/18 thermobalance TGA connected via quartz capillary (in 150 °C jacket) to a Balzers Thermostar quadrupole mass spectrometer set at 70 eV (electron energy). This allowed major ion traces to be obtained (electron impact) along the entire heating program and is presented as mass/charge (*m*/*z*). Whole soils (approximately 40 mg) were suspended in a Pt pan and heated at the same rates as above in a stream of aerobic purge gas flowing at 40  ml/min.

## Results and Discussion

### Typical soil thermal patterns

**Figure 1:**
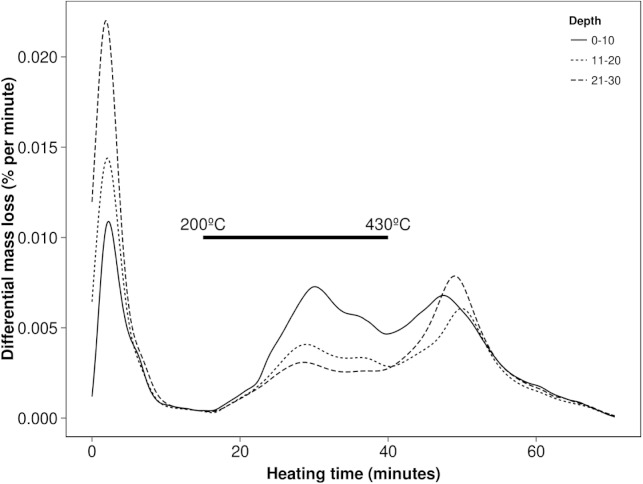
Typical soil thermal characteristics at various depths using the differential mass losses (DTG) obtained by TGA (here of a sandy clay loam at one site).

Plotting the first derivative of mass changes (DTGs) over the heating cycle (time or temperature along *x*-axis) allows separate events to be more readily distinguished and assists in the measurement of their proportions. Using this technique, soils can exhibit thermal characteristics which may be affected by its type, management or sampling depth as shown in Fig. 1. These relative differences seen in the interval 15 and 70 min run time (200–590 °C) are related primarily to the amounts of carbon (200–430 °C) and clay minerals (430–590 °C) producing the typical patterns. For example the trends shown here are easily attributed to the regular changes in texture and OM with depth (Conant, Smith & Paustian, 2003; McKenzie et al., 2004).

The mineral fractions of soils have been widely studied by thermal methods (Fanning, Keramidas & El-Desoky, 1995; Frost & Vassallo, 1996; Hedley, Yuan & Theng, 2007) providing a baseline for underlying inorganic reactions from micas and clays when considering organic constituents within whole soils. Mass changes occurring in the 430–600 °C interval appear to be dominated by the mineral fraction (reflected in Fig. 1) and furthermore when using an inert gas, mass changes in this interval are not noticeably affected indicating decomposition rather than oxidation. By contrast, mass losses over the thermal region 200–430 °C are significantly greater when aerobically purged, consistent with OM. Thermal decomposition of most carbonates is quite separate and obvious from other constituents occurring over 600 °C (Kasozi, Nkedi-Kizza & Harris, 2009) with the exception of more soluble types such as siderite which have much lower decomposition temperature at around 500 °C (Alkac & Atalay, 2008). During thermal analyses this would contribute to (and possibly confound) the changes related to clay minerals but generally these carbonates are less common in soils and do not persist below pH of 9.5 (Rayment & Lyons, 2011). It should be similarly noted that other minerals such as Goethite or Gibbsite which is more common in tropical soils, decompose in the 210–550 °C region (Kloprogge, Ruan & Frost, 2002) along with OM and therefore have the potential to interfere with TGA analyses conducted on whole soils.

### Quantitative TGA and dry combustion

A selection of eastern Australian soils were studied for their TGA behaviour and the principal mass losses quantified. Along with relevant soil information these have been assembled on Table 1 with respective carbon contents found by dry combustion (elemental analysis). The soils are mostly carbonate free and comprise sandy clay loams (Camden, NSW), Dermosols (Hunter Valley, NSW), Vertisols (Liverpool Plains and Narrabri, NSW) and several local soils ranging in texture. The principal mass loss regions were obtained by the natural separations (condition specific) as described by differential mass loss curves or DTGs corresponding to the two temperature bands that correspond to *organic* and *predominantly inorganic* events contained in the 200–430 and 430–590 °C regions respectively (Table 1). Some soils contained carbonates and this was readily measureable using this technique (590–750 °C region).

**Figure 2:**
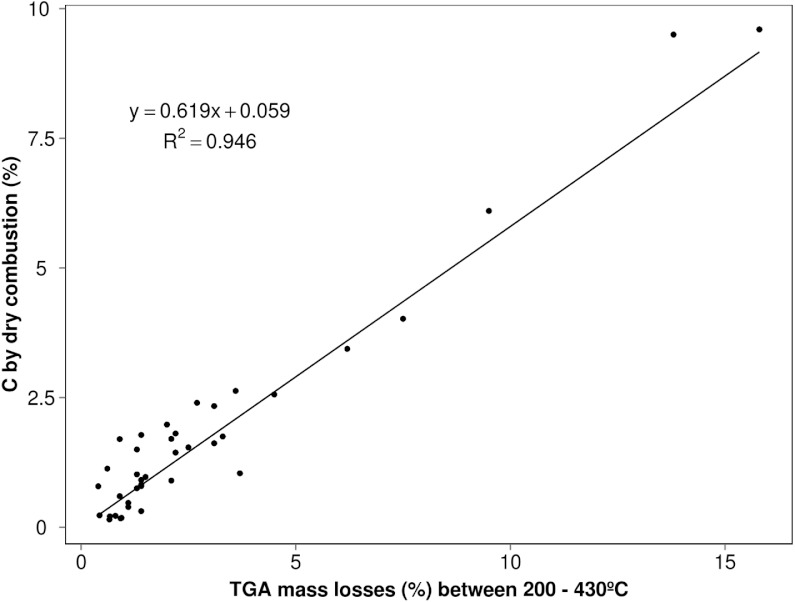
Relationship between TGA mass losses between 200 and 430 °C interval and the total carbon content determined by dry combustion for the soils on Table 1 (relative to van Bemmelen line obtained by multiplying these mass losses by 0.58).

The elemental analyses (%C) obtained for these various study soils (*n* = 39) were most closely correlated (Fig. 2) with mass changes occurring over the 200–430 °C indicating this to be the (most) significant thermal region for SOM release. A slope of 0.62 (*R*^2^ = 0.95) provides an approximate conversion factor for these different soils that is a little higher than the *traditional* factor of 0.58 (van Bemmelen, 1891) and could be suggesting that not all the C is accounted for by this region alone, in those soils. Nonetheless it highlights the most important part of the thermogram for the bulk of OM and is consistent with what can be observed in thermogravimetric soil characterisations under aerobic conditions. While this region probably encapsulates the most labile OM, any shortfall indicated by this comparison is more thermally recalcitrant C such as black carbon (char) and quite likely, equally resistant organic substances tied up with the expected clay mineral event which follows over the 430–590 °C. Very few of our soils have high C contents but removal of the extreme points on Fig. 2 lowers the factor to 0.57 (lowers coefficient of determination to 0.84) but it is anticipated a greater number of high C soils would only reinforce that existing trend. A similar approach taken by Gerzabek et al. (2006) on a small set of soils yielded a value close to 0.59 (mass loss maxima 330 and 440 °C) while Plante et al. (2011) obtained a considerably lower value (0.49) for the slope of regression but the temperature interval included mass losses to 600 °C (possible contribution from other reactions).

There is some deviation or scatter of the data points relative to the calculated (TGA_200–430_ multiplied by 0.58) van Bemmelen line. Any under-bias (under van Bemmelen line) could be attributed to minor structural water from the 320 °C region although, according to Ball (1964) this should be minimal where most of the water loss from clay minerals pre-dried (105 °C) should appear between 450 and 600 °C. Conversely any over-bias (above van Bemmelen line) as most points appear to, is indicative of unaccounted for, more thermally stable C-matter which has not reacted until higher temperatures are reached. Analyses relying on this temperature interval would be relatively diminished by some amount (in comparison to the true value found by dry combustion >600 °C) which could be minor but is an uncertain quantity from soil to soil.

### Investigating the thermal distribution of soil carbon – Evolved gas analysis (EGA) by TGA/MS

**Figure 3:**
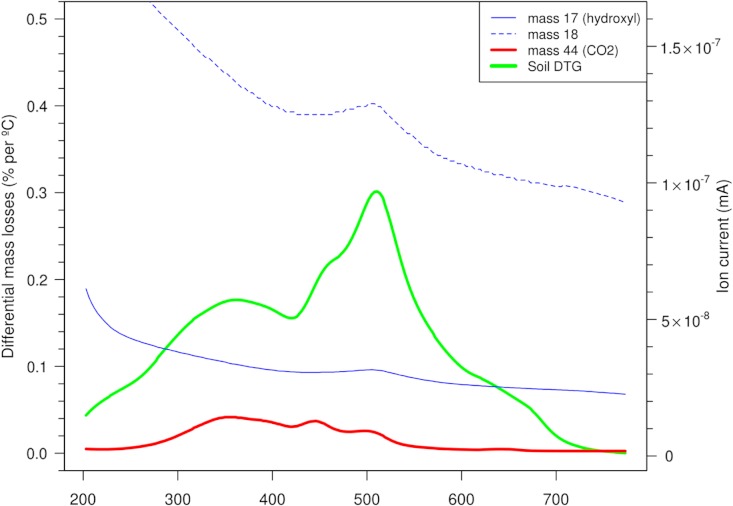
Monitoring ion currents (mass 17, 18 and 44) for oxidised products evolving from a cropped Vertosol aerobically heated by TGA program (10 °C/min between 200 and 800 °C) along with the differential mass changes (DTG). Note the continuation of C release from OM (*m*/*z* 44) beyond 430 °C where inorganic reactions dominate.

To ascertain what reactions/compositions prevail over the TGA heating cycle, combined thermal experiments were conducted on a small number of whole soils. This could uncover decomposition/oxidation and the significance of C-release beyond 430 °C which may be quite variable possibly reflected on Fig. 2. To be able to use *m*/*z* 44 (CO_2_) as a proxy for C release from OM assumes that its relatively low concentrations in whole soils, an excess of oxygen in the purge gas and sufficient active sites ensures quantitative conversion to CO_2_ and water (without charring or recondensing). Major ions were recorded as they evolved into the MS from the programmed heating as mineral material and OM degraded/combusted. The kinetic distribution of carbon (*m*/*z* 44) could now be observed (unlike the previously combined signals from DTGs) which interestingly continues into the temperatures where clays (*m*/*z* 17, 18) degrade as in the example of a Vertisol under crop shown in Fig. 3. The mass changes in the 200–430 °C and then the 430–590 °C region correspond to the destruction of OM and a combination of predominantly inorganic and lesser organic substances respectively. Water and hydroxyl units indicated in the EGAs (*m*/*z* 18, 17) peaked around 500 °C from a falling baseline which follows initial surface dehydration (to 200 °C). Carbonaceous material indicated by *m*/*z* 44 reached a maximum around 350 °C, which was in keeping with the correlation of mass changes over 200–430 °C with dry combustion on Fig. 2. Other soils have shown similar ion patterns where most of the organic material is destroyed (CO_2_ released) over the first thermal region (200–430 °C) with lesser and variable amounts in the second (430 °C+). The *m*/*z* 17 or 18 ion traces did not indicate the presence/breakdown of hydroxide or oxy-hydroxide minerals that would add to mass losses in the thermal region near 300 °C as noted in some soils (Boyle, 2004; McCarty et al., 2010).

These experiments have further demonstrated that OM persists into the temperature regions where clays lose most of their mass, raising this accompaniment between OM and clay or other minerals such as an organo-mineral complex (Oades, 1995; Kleber et al., 2011). Other approaches need to be employed to provide reasonable evidence of the physiochemical linkages between clay and C, often described as a shielding/encapsulation mechanism (Bachmann et al., 2008; Brunn et al., 2008; Flessa et al., 2008; McCarthy et al., 2008) and more lately organic micelles in mineral defects as possible sites (Wilson et al., 2008; Chenu & Plante, 1996). Irrespective of this, the OM is probably more recalcitrant (Plante, Pernes & Chenu, 2005; Plante et al., 2011), may be thermally similar to char particles but degrades at the same temperatures that the mineral components lose mass (i.e. tightly held water/hydroxyl units) making it difficult to quantify by simple LOI methods.

### The relevance of grain size and implications for loss-on-ignition (LOI) methods

**Figure 4:**
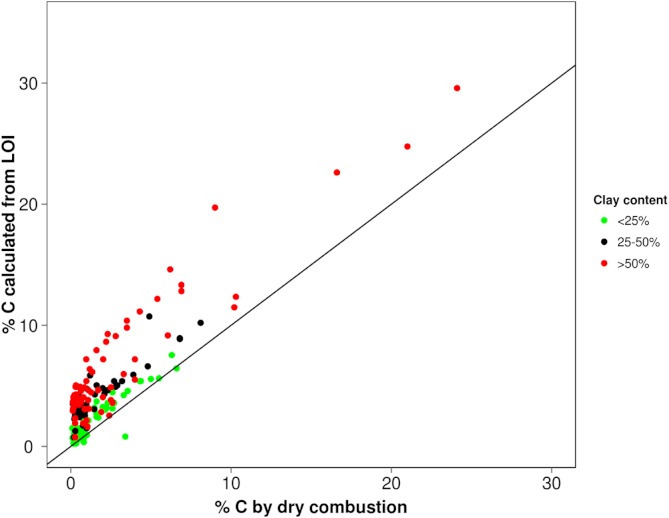
Correlation of LOI derived % C (using 0.58) with their values determined by dry combustion relative to 1:1 line.

**Figure 5:**
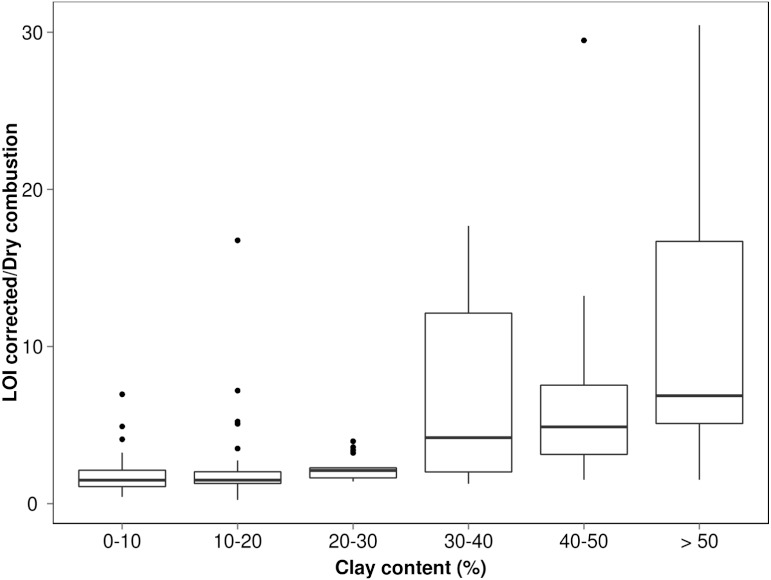
Box plot measuring relationship between soil C by LOI and soil C by elemental analysis for each clay class where ends of the boxes are 25th and 75th quantiles and mid-line the median value.

What does this merging of residual SOM and mineral matter mean for LOI determinations? To explore and demonstrate any possible pattern of C-overestimation with grain size, we referred to a legacy database where there was sufficient corresponding dry combustion, LOI and textural data (*n* = 208) (acquired with methods described by Rayment & Lyons, 2011). The % carbon values derived from LOI (using factor 0.58) were plotted against dry combustion (elemental analysis) but separated into <25, 25–50 and >50% clay to highlight these differences (Fig. 4). To obtain greater statistical detail about how the calculated values deviate from the *true* value (expressed here as %C LOI/%C dry combustion) for the respective textural classes, these have been presented as a box plot on Fig. 5. Clearly the coarser textured soils tend towards parity while the more clay-rich (especially >30%) soils show a greater variability in this relationship indicating quite large overestimation in some cases.

**Figure 6:**
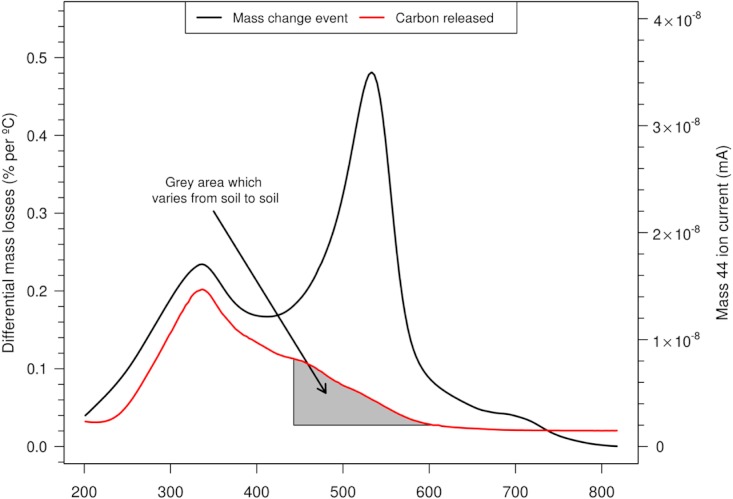
Differential mass loss (DTG) and C-trace (*m*/*z* 44) for Vertisol under pasture to assess benefit of LOI temperatures over 430 °C which are detailed on Table 2.

To get some real perspective of how much C is unaccounted for when applying more conservative LOI temperatures or conversely how much erroneous mass is included at higher temperatures, these relative amounts have been assessed using the coupled TGA/MS approach. In the example on Fig. 6 of a Vertisol (under pasture) corresponding ion currents were plotted along with mass changes represented by the DTG. These initially track the differential mass loss curve but deviate beyond about 430 °C enabling a relative quantification of how much C remains using the *m*/*z* 44 ion as all OM is released through combustion. The additional mass losses beyond this temperature which derive primarily from increasing inorganic reactions provides a guide of how much unrelated mass loss needs to be incurred in order to obtain those diminishing increments of OM (i.e. %C). The data on Table 2 (determined using LoggerPro, Vernier Software and Technology) shows a benefit – sacrifice regimen to assess how worthwhile each subsequent temperature interval is in terms of returning these extra few %C (over and above the 3.46% mass due to only OM). Obviously this varies considerably depending on the soil texture and OM complexity/type. Interestingly, Siewert (2004) correlated principal soil components with mass changes up to 1000 °C in 10° steps. Very significantly C and N were strongest in the range 200–500 °C and clay % increased to 550 °C producing a composite plot (correlation coefficients against temperature) showing some resemblance to the ion plots in this study.

**Table 2:** Increments of % C (OM) and % mass loss (mostly from mineral) for intervals beyond 430 °C.

Temperature (°C)	% of total C	Additional % mass loss	Cumulative % mass loss
440	76.5	0.20	3.66
460	81.0	0.39	4.05
480	87.0	0.48	4.53
500	91.3	0.61	5.14
520	95.0	0.84	5.95
540	96.9	0.94	6.89
560	98.9	0.52	7.41

LOI temperatures vary between laboratories (Rayment & Lyons, 2011 and their sources; Sutherland, 1998) and in many cases are likely to overestimate OM values where temperatures are excessive. It is expected that LOI would yield reliable C determinations on sandy or peaty soils and not so much for clay-rich soils which may explain previous concerns over the method (Leeper & Uren, 1993; Sutherland, 1998; Heiri, Lotter & Lemcke, 2001). However it is suggested that the method should be fairly reliable provided they adhere to careful heating regimes and observe the mass changes between 200 and 430 °C which should exclude changes from all inorganic reactions (dehydration and clay collapse). Generally the conversion factor of 0.58 has been regarded by many as not universally applicable due to heterogeneities between soils and should be refined for each area or soil type (Sutherland, 1998; Kasozi, Nkedi-Kizza & Harris, 2009; Pribyl, 2010). In their summary on carbon determination methods, Chatterjee et al. (2009) indicate 430 °C (similarly conservative temperatures suggested by Nelson & Sommers, 1996) as a recommended LOI temperature after drying the soil at 105 °C which should, based on our work, yield a reasonably good estimate of OM and hence TOC. However it can be noted that it excludes any variable amounts of more resistant OM oxidisable at higher temperatures such as charred particles or what might possibly be tied up with the mineral component. TGA combined with other detection techniques clearly reveal the overlap of CO_2_ due to organic matter and OH^−^ from inter-crystalline losses which could give rise to overestimation when mass-only changes are considered (as in LOI beyond temperatures of 430 °C). Conversely, the proportion of residual OM (overlapping with inorganic mass changes) that would be excluded would result in small underestimations but should provide greater accuracy than yields from higher temperature as is often the practice.

It is herein proposed that the C distributed in these two main thermal bands could be determined separately by elemental analysis on duplicate soils by oxidising at 430 and then again at 550 °C, the difference in C content between the two, potentially representing the more stable portion. Further studies to map the thermal distribution of C in diverse soils, depths and land-uses by either TGA/MS or TGA/FTIR is encouraged to uncover any relationships with OM maturity or the presence of other C forms such as biochars or those that may be mineral bound. Such combined analyses assist in the interpretation of mass change events where whole soils can contain numerous constituents such as iron or aluminium oxides and clays minerals as well as OM.

## Conclusions

Thermal analytical techniques have been a useful tool in elucidating the distribution of carbonaceous materials in soils. They have exposed some of the weaknesses of LOI methods but on the other hand serve an excellent guide to allow its more reliable application. It has demonstrated that care needs to be exercised when applying LOI with an awareness of the relevant thermal regions and reactions of the various soil components as seen using TGA and adjunct techniques. The soil data from this study has indicated that traditional conversion factors may provide a reasonable carbon estimate for finer textured soils using LOI methods as long as temperatures are constrained between 200 and 430 °C. Data acquired using higher temperature methods (e.g. 550 °C) would require more intensive site-specific calibration which should automatically factor in textural variations.

Thermal methods readily divide organic and inorganic C, as is well known, but could potentially be used to distinguish other kinetic pools such as biochars which make it well suited to the assessment of management practices for C sequestration. Further work into the carbonaceous material associated with different particle size fractions and mineral matrices could be very valuable also.
